# Validation of a method evaluating T cell metabolic potential in compliance with ICH Q2 (R1)

**DOI:** 10.1186/s12967-020-02672-7

**Published:** 2021-01-06

**Authors:** Patricia Mercier-Letondal, Chrystel Marton, Yann Godet, Jeanne Galaine

**Affiliations:** 1 Univ. Bourgogne Franche-Comté, INSERM, EFS BFC, UMR1098 RIGHT Interactions Greffon-Hôte-Tumeur/Ingénierie Cellulaire et Génique, 25000 Besançon, France; 2 Etablissement Français du Sang Bourgogne Franche-Comté, Activité Médicaments de Thérapie Innovante, 25000 Besançon, France

**Keywords:** T cell based immunotherapies, T cell metabolic potential, Control material

## Abstract

**Background:**

Metabolic cell features are able to give reliable information on cell functional state. Thus, metabolic potential assessment of T cells in malignancy setting represents a promising area, especially in adoptive cell therapy procedures. Easy to set up and convenient Seahorse technology have recently been proposed by Agilent Technologies and it could be used to monitor T cells metabolic potential. However, this method demonstrates an inter-assay variability and lacks practices standardization.

**Results:**

We aimed to overcome these shortcomings thanks to a lymphoblastic derived JURKAT cell line seeding in each experiment to standardize the Seahorse process. We used an adapted XF Cell MitoStress Kit protocol, consisting in the evaluation of basal, stressed and maximal glycolysis and oxidative phosphorylation related parameters, through sequential addition of oligomycin and carbonyl cyanide 4-(trifluoromethoxy)phenylhydrazone (FCCP) to a glucose containing medium. Data were acquired and analyzed through Agilent Seahorse XFe96 analyzer. Indeed, we validated this method in the light of ICH Q2 (R1) guidelines. We were able to confirm the specificity and accuracy of the method. We also demonstrated the precision, linearity and range of the method in our experimental conditions.

**Conclusion:**

The validation of the method consisting in a JURKAT cell line experimental incorporation as a control material contributes to improve the Seahorse technology’s robustness. These results lay the groundwork for the implementation of this technology to optimize T cell based cellular therapy products production process and monitoring.

## Background

Metabolic pathways represent the way for cells to ensure their own energy synthesis, their proliferation-required biosynthesis and to maintain an adequate red/ox potential. These pathways are able to consume a multiplicity of nutrients, such as glucose, glutamine or fatty acids, and are interconnected to reach these survival-directed objectives. Among them, oxidative phosphorylation (OXPHOS) and glycolysis have been extensively studied in the last decades. First, they have been evaluated in the setting of metabolic diseases, such as type II diabetes [1, 2]. Then, other fields have addressed the differential cell resort to these pathways. Effects of drugs on metabolism have been indeed studied [3], as well as relationship between pathology-induced dysregulations and metabolic functions [4–6]. In the field of cancer biology and therapy, metabolic potential assessment and management represents a promising area. On the one hand, metabolic phenotype of cancer cells is interesting as it potentially represents an antitumor therapeutic target. Indeed, cancer cells have the peculiarity to consume glucose via anaerobic glycolysis even in presence of oxygen. This phenomenon is known as “Warburg effect” and allows cancer cell to fulfill bioproduction needs in order to sustain intense proliferation rather than energy production [7]. As a consequence, tumor microenvironment is especially glucose-depleted and lactate-enriched. Even if mammalian Target Of Rapamycin (m-TOR) pathway has ambivalent effects on T cell anti-tumor capacities [8, 9], m-TOR inhibitors as glucose metabolism targeting chemotherapies [10] could make glucose available in tumor microenvironment for T cells by reducing cancer cell glucose consumption, thus taking part to limit T cell exhaustion [11]. On the other hand, either myeloid or lymphoid immune cells are described to have specific metabolic signatures [12] which potentially translate into functional fate [13]. The latter is a topical issue regarding the characterization of T cell functionality and fitness, especially for T cell based therapies procedures such as tumor infiltrating lymphocytes, chimeric antigen receptor (CAR) or TCR-transgenic T cell infusions. Furthermore, T cell metabolism status could potentially be driven and monitored during the ex vivo expansion step of T cell based therapies procedure, in order to potentiate the anti-tumor immune response and effector T cell post infusion persistence [10, 11, 14].

Different methods are available to evaluate the capacity of OXPHOS and glycolysis to meet metabolic requirements. Among them, non-destructive methods enable the evaluation of the effect of several compounds impacting energetic metabolism pathways. First, methods carrying out individual metabolites or nutrients assays, such as glucose, lactate or adenosine triphosphate (ATP), are easy to use, convenient, relatively cheap and generally non-destructive. However, these are poorly informative on the cell global metabolic status [15, 16]. Moreover, the main inherent failure in ATP assays remains the difficulty to distinguish OXPHOS from glycolysis involvement [17]. Other metabolomic profiling methods by nuclear magnetic resonance spectroscopy and/or mass spectroscopy represent powerful and accurate tools to assess the relative cell resort to metabolic pathways. Nevertheless, these approaches require specific competences and equipment, remain expensive and do not provide a global snapshot of metabolic pathways [15, 16]. Metabolic flux measurements are valuable methods to evaluate cell metabolic requirements: some of them involve radioactive substrate, difficult to implement even if highly specific; others involve phenotypic arrays, such as Biolog system (Phenotype MicroArrays™), permitting a real time assessment of the flux although requiring heavy bioinformatic analysis and does not allow the observation of perturbations [16]. The activity evaluation of rate-limiting enzymes, specifically those related to metabolic pathways, is another alternative to study cell metabolism. This kind of experiment is easy-to-use and relatively costless, but requires cell destruction, thus preventing kinetic analysis or modulation [15]. Several methods based on medium acidification detection are available and involve either cumbersome and low-throughput electro-chemical means, or more or less accurate pH-sensitive probes [18]. Cellular oxygen consumption, an OXPHOS-related parameter, can be determined by a myriad of methods. Indeed, electron paramagnetic resonance detection methods allow for easy and continuous measurements when coupled to O_2_ sensitive probes, but is expensive, complex and one-shot-related [16]. Polarographic method involving a Clark oxygen electrode is reliable, but needs a substantial amount of biological material and is often cell destructive, even if permeabilized cells can be used [19]. O_2_-quenched fluorescent probes exist and allow for an accurate and non-destructive assay of cellular oxygen consumption. Recently, Seahorse XF analyzers were developed by Agilent Technologies. They include an O_2_-quenched and a pH-sensitive fluorescent probes and are able to assess both cellular oxygen consumption and extracellular acidification. The method is non-destructive and a few amount of biological material is required for its implementation. The design of the assay enables a real-time sensitive kinetic evaluation of Oxygen Consumption Rate (OCR) and Extra-Cellular Acidification Rate (ECAR) key parameters, both at steady state and after compound exposure. Indeed, up to four metabolic pathway-impacting-compounds can be added in each microplate well, allowing for distinguishing OXPHOS from glycolysis cell resort. It is important to note that OCR measurement is commonly associated with OXPHOS, and that ECAR is considered as a robust indicator of glycolysis. Nevertheless, medium acidification can also be mediated by several other proton producing pathways, such as OXPHOS. According to Konrad et al. [20], a baseline cellular OCR/ECAR ratio < 4 indicates that CO_2_ production represents a negligible contribution to ECAR. This metric is likely to reduce the bias in ECAR interpretation.

Briefly, a multi-well culture plate with seeded-cells is used. This plate is capped by a sensor cartridge having two embedded fluorophores: one is quenched by extracellular dissolved O_2_ and the other one is sensitive to extracellular free proton concentration. The signal emitted by these probes is transmitted via optic fiber bundle to the Seahorse analyzer, and given OCR and ECAR values are automatically calculated. The sensor cartridge is able to lift into the well in order to create a low cellular medium volume transient micro-chamber, allowing for a measurement of key parameters in a highly sensitive and accurate way. A loading guide facilitates the addition of at least four metabolism impacting compounds. Many of them are available to evaluate which metabolic pathway is promoted by cells and to define cellular subset metabolic signatures. The method is easy to set up in every lab with few competences in metabolic pathways, in order to compare metabolic phenotype of multiple cell subsets. Nevertheless, it is important to keep in mind that cell culture conditions and seeding parameters need to be controlled in order to get reliable data. Indeed, cell culture conditions rely on cellular fitness, which can be impacted by nutrients availability or acidification level in the medium, and on culture confluence before Seahorse cell plating. Further, seeding parameters depend on cell counting, quality of cellular attachment or cell confluence in the Seahorse plate wells.

Despite these many claims, the inter-assay variability of the Seahorse method is a well-described issue [21]. The analysis of experiments performed through distinct plates and acquired at different times can be compromised. Indeed, the comparability of inter-assay obtained data is difficult to reach. Yépez et al*.* [21] already showed that between-plate variation largely dominates within-plate variation. Overcoming this shortcoming could represent a way to improve the robustness of the method and make it a new gold standard, even a potential Good Manufacturing Practices (GMP)-compliant validated method for metabolism studies, in the setting of quality control and monitoring of T cell based therapies productions. Furthermore, Yépez et al*.* [21] raised the issue of lacking best practices for Seahorse run design and analysis, despite plethoric literature available about Seahorse experimental aspects related to assay preparation.

This lack of robustness could be improved by implementing an Internal Quality Control (IQC) process. IQC process consists in inserting one or more control materials into each run of analysis. The control materials are treated by an analytical procedure identical to that performed on the test materials. The essential properties of control materials are homogeneity and stability, in order to avoid method drift over time. This may mean that the control material can be different and behaves slightly differently from sample [22]. In this way, our study aims to control inter-assay variability of Seahorse technology in the setting of the quality control and monitoring of T cell based therapies products by using a JURKAT tumor cell line as an IQC process-associated control material. JURKAT cell line is a human T-leukemic cell line suitable to mimic cultured T cell behavior. Moreover JURKAT cells contribution of glycolysis to proton efflux rate is around 90% [23]. Actually, primary T cells are inherently heterogeneous and show high inter-individuals variability, whereas JURKAT cell line is homogeneous and stable insofar as its culture conditions are tightly monitored. Thereby, the number of passages has to be checked as well as the log phase of the propagation has to be met to ensure optimal stability of the control material [24]. To do so, method validation criteria were evaluated in the light of requirements of the International Council Harmonization (ICH) Q2 (R1) [25] guidelines. These guidelines are dedicated to analytical method validation in order to provide evidence that the method is suitable for its intended purpose. It is important to note that this kind of analysis is non-compendial and should be performed in the setting of investigational Advanced Therapy Medicinal Products (ATMPs).

## Results

### Assay design and impact on metabolic potential analysis

**Fig. 1:**
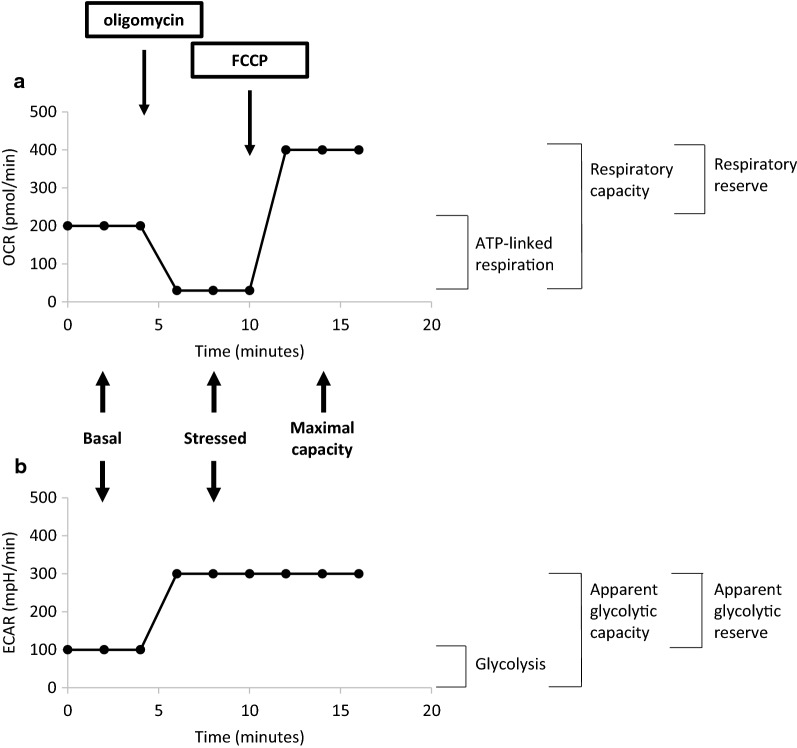
Assay design and impact on metabolic potential analysis.** a** Cartoon illustration of OCR levels versus time at baseline, after oligomycin and after FCCP injections. **b** Cartoon illustration of ECAR levels versus time at baseline, after oligomycin and after FCCP injections

It was considered that sufficient metabolic potential related information were displayed using glucose-containing culture medium at steady state, after adding oligomycin in the port A and carbonyl cyanide 4-(trifluoromethoxy)phenylhydrazone (FCCP) in the port B of the Seahorse analyzer plate. Sequential addition of these two compounds corresponds respectively to stressed-metabolic condition and metabolic maximal capacities. Oligomycin inhibits the ATP-synthase resulting in disruption of mitochondrial ATP production and causes an ATP-linked respiration breakdown and a subsequent increased glycolysis cell resort in order to meet the cellular energy requirement. FCCP uncouples oxygen consumption from ATP production, restores the mitochondrial membrane potential because of depolarizing this membrane, leading to the maximization of OXPHOS. Indeed, observed difference between basal and oligomycin-induced OCR and between FCCP-induced OCR and basal, and FCCP-induced OCR and oligomycin-induced OCR represents respectively ATP-linked cell respiration, respiratory reserve and respiratory capacity (Fig. 1a, inspired by Divakaruni’s analysis [26]). Moreover, the observed difference between oligomycin-induced and glucose-enriched basal ECAR can be assimilated to apparent glycolytic reserve; similarly, the total ECAR observed after oligomycin exposure can be assimilated to apparent glycolytic capacity (Fig. 1b, diagram derived from Mookerjee’s work [27]).

### Determination of optimal JURKAT cell line concentration and specificity of the method

**Fig. 2:**
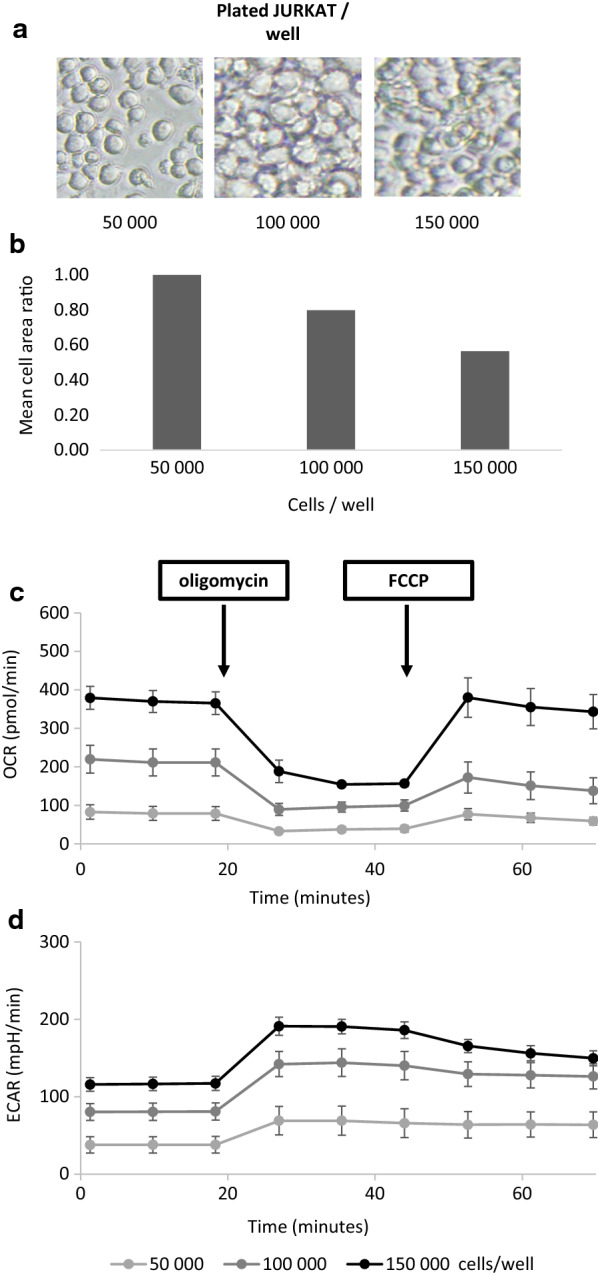
Determination of optimal JURKAT cell line concentration and specificity of the method.** a** Optical images of JURKAT cell confluence. JURKAT cells were plated at 50,000, 100,000 or 150,000 cells/well in a 96-well plate and photographed after poly-D-lysine cell attachment. **b** Ratio of cell area on cell area obtained in the 50,000 cells/well plated condition (ten area measurements per cell seeding condition). **c** Illustration of OCR levels versus time at baseline, after oligomycin and after FCCP addition, for cell concentration of 50,000 (light grey line), 100,000 (dark grey line) and 150,000 (black line) cells/well, 6 technical replicates per condition, each point corresponds to mean ± standard deviation (SD) of replicates values, this is one experiment representative of 4 independent experiments. **d** Illustration of ECAR levels versus time at baseline, after oligomycin and after FCCP addition, for cell concentration of 50,000 (light grey line), 100,000 (dark grey line) and 150,000 (black line) cells/well, 6 technical replicates per condition, each point corresponds to mean ± SD of replicates values, this is one experiment representative of 4 independent experiments

Seahorse analysis was performed using different numbers of JURKAT cells per culture well: 50 000, 100,000 and 150,000. Each condition was performed in 6 technical replicates. First, pictures of cell-held plastic support were took (Fig. 2a). A dose of 50,000 cells/well allowed for a 50–60% confluence. Doses of 100,000 and 150,000 cells/well allowed for 90–100% confluence. It’s noteworthy that the more concentrated cells were, the less important the cell area was (Fig. 2b). Basal, stressed and maximal OCR (Fig. 2c) plus basal and stressed ECAR (Fig. 2d) obtained with different amounts of JURKAT cells concentrations were evaluated. As expected, oligomycin injection resulted in OCR values breakdown and in ECAR values increase. Similarly, FCCP exposure caused a potentiation of OCR values and had no substantial effect on ECAR values. These data, obtained with JURKAT cell line matrix, confirmed the specificity of the assay in line with the expected OCR and ECAR variations induced by oligomycin and FCCP. Impurities could have been present in the cell environment and have biased specificity assertion. Culture medium composition, in term of nutrients content and pH, was monitored extemporaneously before performing experiment and should not be likely to interfere with cell metabolic activity detection. Poly-D-lysine residues potentially released in the medium could also interfere with OCR and ECAR evaluation. However, it is highly unlikely because data were automatically corrected by the Seahorse system using four-edge calibration wells and were equal to 0.00 for every performed experiment (n = 4, data not shown). For all three sequential steps, a dose–response between cell concentration and OCR and ECAR measurement was observed. The higher the cell concentration was, the higher the OCR and ECAR values were. This proportionality relationship would not be prone to arise in case of impurities interference. Altogether, these elements contributed to specificity assessment. Basal OCR/ECAR ratio obtained for 50,000, 100,000 and 150,000 JURKAT cells/well are 2.12, 2.66 and 3.19, respectively. All these three values are inferior to 4, allowing us to conclude that contribution of OXPHOS to ECAR is negligible. Further, it is important to note that FCCP did not have consequent impact on ECAR in this experimental setting, underlying a non-substantial contribution of OXPHOS to medium acidification. For the following experiments, a concentration of 100,000 JURKAT cells/well was chosen in accordance with Agilent’s database.

### Accuracy of the method

**Fig. 3:**
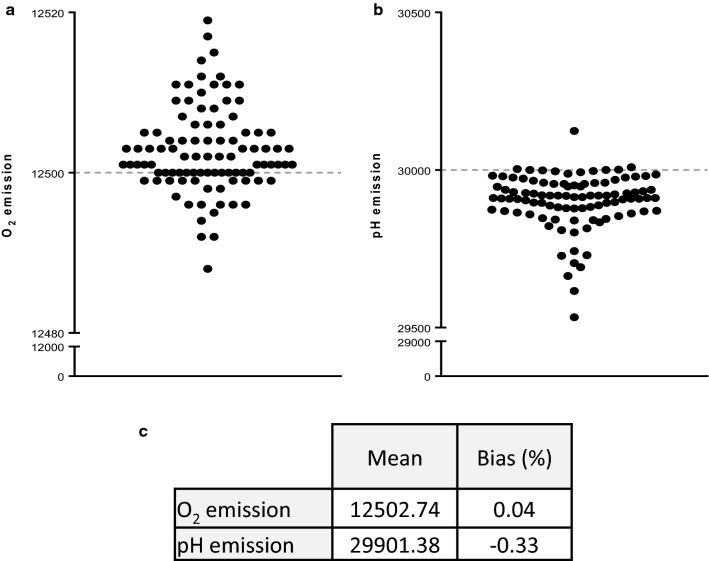
Accuracy of the method.** a** Distribution of O_2_ emission values 96 replicates (black full circles), around target value equal to 12,500 (disrupted grey line). **b** Distribution of pH emission values 96 replicates (black full circles), around target value equal to 30,000 (disrupted grey line). **c** Metrics table associated with O_2_ emission and pH emission: mean and bias, expressed in percentage, are shown

Seahorse technology implies a systematic calibration of the equipment by measuring OCR and ECAR of Calibrant buffer containing wells, using the utility plate. Raw data concerning this calibration step are provided for every experiment by Wave software. This calibration operation is able to provide data related to accuracy of the method, even if it is not possible to confirm this validation criteria in the setting of the use of JURKAT cell line. Nevertheless, method bias obtained in the setting of Seahorse calibration assessment of O_2_ (Fig. 3a) and pH (Fig. 3b) emission values are 0.04 and − 0.33%, respectively, for 96 replicates (Fig. 3c). These bias values are largely less than 5%, confirming the excellent accuracy of the detection of O_2_ consumption and medium acidification.

### Precision of the method

**Fig. 4:**
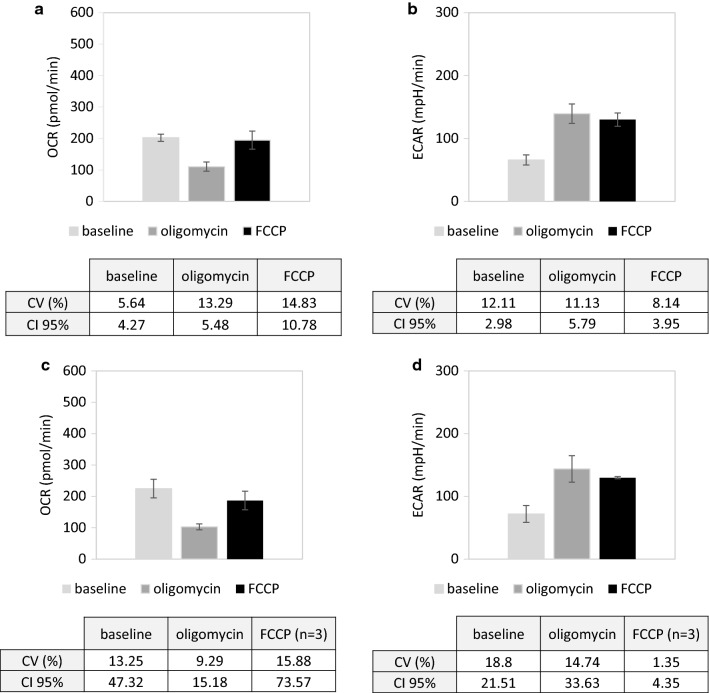
Precision of the method.** a** Repeatability assessment of OCR detection. Left: illustration of mean ± SD of OCR levels (10 technical replicates, 3 times of measurement) at baseline (light grey bar), after oligomycin (dark grey bar) and after FCCP addition (black bar) (left). Right: table of statistical metrics at baseline, after oligomycin and after FCCP addition. CV: coefficient of variation, expressed in percentage; CI 95%: confidence interval. **b** Repeatability assessment of ECAR detection. Left: illustration of mean ± SD of ECAR levels (10 technical replicates, three times of measurement) at baseline (light grey bar), after oligomycin (dark grey bar) and after FCCP addition (black bar).Right: table of statistical metrics at baseline, after oligomycin and after FCCP addition. CV: coefficient of variation, expressed in percentage; CI 95%: confidence interval. **c** Intermediate precision assessment of OCR detection. Left: illustration of mean ± SD of OCR levels (4 independent experiments for baseline and oligomycin addition, 3 independent experiments for FCCP addition) at baseline (light grey bar), after oligomycin (dark grey bar) and after FCCP addition (black bar).Right: table of statistical metrics at baseline, after oligomycin and after FCCP addition. CV: coefficient of variation, expressed in percentage; CI 95%: confidence interval. **d** Intermediate precision assessment of ECAR detection. Left: illustration of mean ± SD of ECAR levels (4 independent experiments for baseline and oligomycin addition, 3 independent experiments for FCCP addition) at baseline (light grey bar), after oligomycin (dark grey bar) and after FCCP addition (black bar). Right: table of statistical metrics at baseline, after oligomycin and after FCCP addition. CV: coefficient of variation, expressed in percentage; CI 95%: confidence interval

First, the repeatability of the method in terms of OCR (Fig. 4a) and ECAR (Fig. 4b) was assessed by performing 10 technical replicates of 100,000 JURKAT cells/well. For OCR and ECAR evaluation, every step of the experiment, either basal, stressed or maximal demonstrated a coefficient of variation (CV) inferior to the cut-off value of 15%. Similarly, 95% confidence intervals for these different evaluated key parameters were reduced to achieve values less than 6% of total average values. Taken together, these data assessed the good repeatability of the method. Second, the intermediate precision of the method was determined, through the realization of respectively 4 independent experiments for the assessment of basal and stressed OCR and ECAR, and 3 independent experiments for assessment of FCCP effect on metabolic potential of JURKAT cell line (Fig. 4c, d). Basal and stressed OCR as stressed ECAR showed CV inferior to 15%, which represents the threshold for a good precision. Either maximal OCR or basal ECAR demonstrated respectively CV values of 15.88 and 18.8, largely inferior to the cut-off value of 30%, beyond which the precision is considered as non-acceptable. Ninety-five % confidence intervals are quite high, achieving values up to 39% of the total average values. Nevertheless, we concluded that the intermediate precision of the method was acceptable. It is important to note that the parameter “effect of FCCP treatment on ECAR” showed precision metrics as acceptable as those of others evaluated parameters, even if it did not contribute to our biological analysis.

### Linearity and range of the method

**Fig. 5:**
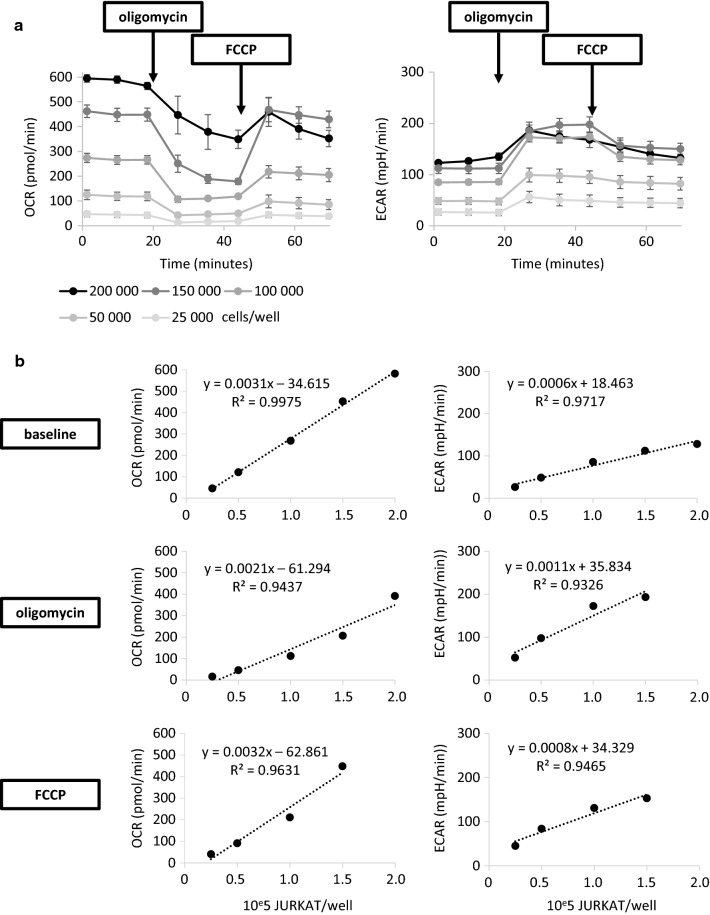
Linearity of the method.** a** Illustration of OCR levels (up) and ECAR levels (down) versus time at baseline, after oligomycin and after FCCP addition, for cell concentration of 25,000 (very light grey line), 50,000 (light grey line), 100,000 (middle grey line), 150,000 (dark grey line) and 200,000 (black line) cells/well, 6 technical replicates per condition, each point corresponds to mean ± SD of replicates values. **b** Illustration of OCR levels (up) or ECAR levels (down) versus cell/well concentration at baseline (left), after oligomycin addition (middle) and FCCP addition (right). Each point represents the mean of 6 replicates values and 3 step measurements. The regression line (disrupted black line) is derived from sum of minus squares method and its metrics (equation and r^2^ value) are represented

In order to evaluate method’s linearity, Seahorse analysis was performed using 5 increasing concentrations of JURKAT cells per culture well: 25,000, 50,000, 100,000, 150,000 and 200,000. Every cell culture conditions were performed in 6 technical replicates. OCR and ECAR were determined and results allowed us to ascertain that, for each evaluated key parameter, a cell dose dependent response was detectable, except in case of 200,000 JURKAT cells/well condition (Fig. 5a). In this last setting, response to FCCP exposure achieved the same level of OCR values than those obtained for 150,000 JURKAT cells/well. Similarly, oligomycin exposure resulted in equal ECAR values between these two conditions. Moreover, basal OCR/ECAR ratio for the dose of 200,000 cells/well was over 4, limiting the analysis of ECAR as a relevant glycolysis indicator. It is noteworthy that in case of 200,000 JURKAT cells/well distribution, standard deviations observed after either oligomycin or FCCP injection were higher that previously demonstrated during method’s precision studies, probably due to over-confluence of cells which cannot correctly attach to the plastic support. We determined for every key parameter the maximal area within which we observed r^2^ value of the regression line superior to 0.92 (Fig. 5b). Metrics parameters of these regression lines were indicated. Among them, γ-intercept observed for basal OCR and ECAR statement was − 34.615 and 18.463 respectively. The maximal common linear area for every key parameter was assessed and corresponded to doses from 25,000 to 150,000 JURKAT cells/well. The dose of 200,000 cells/well was then excluded of the linear area in line with previous observations.

**Fig. 6:**
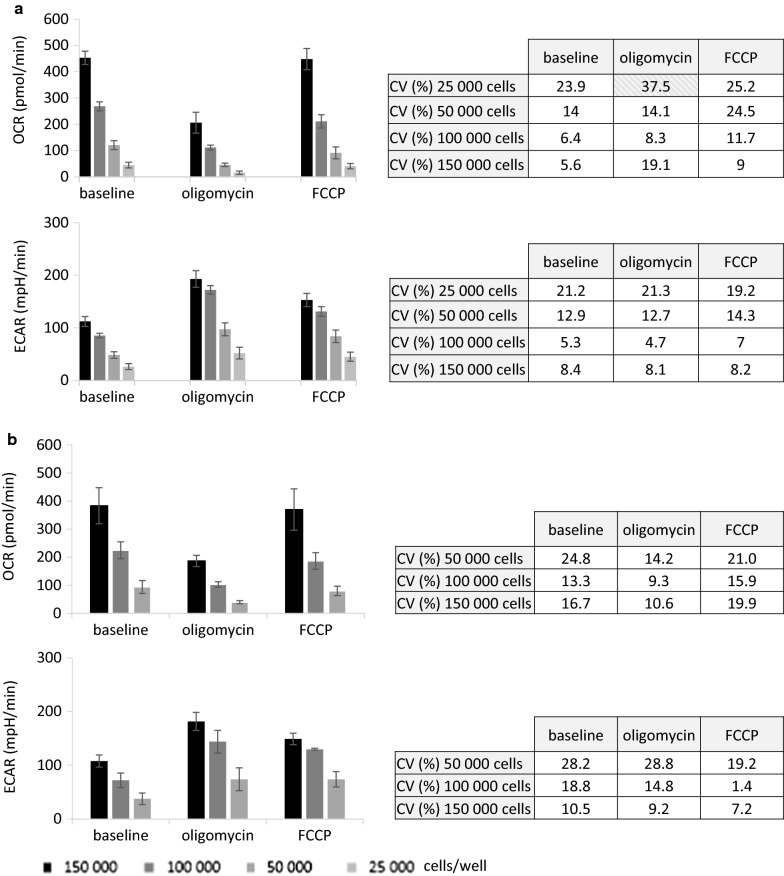
Range of the method.** a** Repeatability assessment of OCR or ECAR detection within the linear area: illustration of mean ± SD of OCR (up) and ECAR (down) levels (6 technical replicates, three times of measurement) at baseline, after oligomycin and after FCCP addition, cells/well concentration of 25,000 (very light grey bar), 50,000 (light grey bar), 100,000 (dark grey bar) and 150,000 (black bar), associated table of statistical metric at baseline, after oligomycin and after FCCP addition. CV: coefficient of variation, expressed in percentage. **b** Intermediate precision assessment of OCR or ECAR detection within the linear repeatable area: illustration of mean ± SD of OCR (up) and ECAR (down) levels (average values of 3 independent experiments) at baseline, after oligomycin and after FCCP addition, cells/well concentration of 50,000 (light grey bar), 100,000 (dark grey bar) and 150,000 (black bar), associated table of statistical metric at baseline, after oligomycin and after FCCP. CV: coefficient of variation, expressed in percentage

Within the maximal common linear area previously identified, range with appropriate repeatability and intermediate precision was determined. First, repeatability was assessed for doses of 25,000 to 150,000 JURKAT cells/well. Each condition was performed in 6 technical replicates (Fig. 6a). Concerning OCR measurements, for doses of 100,000 and 150,000 cells, CV obtained for basal, post-oligomycin and post-FCCP exposures were all less than 15%. For dose of 50,000 cells/well, CV at baseline and after oligomycin injection were also less than 15%. Key parameter in these conditions demonstrated then a good repeatability. For dose of 50,000 cells/well, CV calculated after FCCP adding was comprised between 15–30%. For dose of 25,000 cells/well, basal and post-oligomycin CV achieved values was also comprised between 15–30%. Repeatability of these evaluated points was considered as acceptable. For dose of 25,000 cells/well after oligomycin addition, CV calculation resulted in value superior to 30%, considered as unacceptable in the setting of repeatability validation. For ECAR measurements, all key parameters evaluated for 50,000, 100,000 and 150,000 cells/well resulted in CV less than 15% demonstrating a good repeatability. For 25,000 cells/well concentration, CV values were less than 30%, then the repeatability is acceptable in this condition. Intermediate precision were then determined for doses of 50,000, 100,000 and 150,000 cells/well through the analysis of 3 independent experiments (Fig. 6b). OCR measurements showed good intermediate precision in case of oligomycin treatment of 50,000 and 150,000 cells/well, and in case of basal and post-oligomycin treatment of 100,000 cells/well. Acceptable intermediate precision was assessed in case of basal and post-FCCP treatment of 50,000 and 150,000 cells/well, and of post-FCCP exposure of 100,000 cells/well. ECAR values obtained allowed us to conclude that the intermediate precision was good in the setting of all parameters evaluated for 150,000 cells/well and for post-oligomycin and FCCP treatment for 100,000 cells/well. In the same manner, intermediate precision was considered as acceptable for all parameters of 50,000 cells/well concentration and for basal state of 100,000 cells/well. Altogether, these results permitted us to determine the range within the linear area having an acceptable to good precision, i.e. from 50,000 to 150,000 cells/well.

## Discussion and conclusion

Metabolic pathways analysis gains considerable interest in the field of immunology because of its impact on immune cell effector function. Seahorse technology makes these analysis easy to implement in every lab, as soon as it is provided with the equipment. However, many critical environmental parameters can affect data reliability and including appropriate experimental controls is critical to be confident in scientific results. In this article, we described a simple procedure to standardize metabolic analysis using XFe96 Seahorse analyzer.

We sought to validate a method involving JURKAT cell line as a control material for T cell metabolic potential assessment according to ICHQ2 (R1) guidelines. In this setting, we confirmed that the optimal concentration of JURKAT, in our setting, is 100,000 cells/well, corresponding to an about 90% cell confluence, as recommended by Agilent Technologies and Plitzko et al*.* [28]. The basal OCR and ECAR values obtained for this concentration are comprised between 100 and 300 pmol/minutes and greater than 20 mpH/minutes respectively. These values are considered as acceptable by Divakaruni et al. [26] and TeSlaa et al. [15]. The γ-intercept we obtained for basal ECAR during method linearity studies was around 18, strenghtening TeSlaa’s statement. For this concentration, basal OCR/ECAR ratio is less than 4, allowing us to conclude that ECAR parameter is mainly glycolysis related. This conclusion is supported by the limited effect of FCCP on ECAR measurement. Nevertheless, it should be kept in mind that the relative contribution of respiration and glycolysis to ECAR is likely to depend on cellular type, evaluated cell lines respiratory proton production rate ranging from about 9 to 76% [29].

OCR and ECAR profiles obtained for JURKAT cell line at basal state and after oligomycin and FCCP exposure are in accordance with expected profiles, given the mechanism of action described for these two compounds. These profiles are also in accordance with those described in other models [28]. It is well known that phenotypically less differentiated and less activated T cells showed a more OXPHOS-dependent basal metabolism and a minority resort to glycolysis than more differentiated and activated T cells [30]. Concomitantly, our team realized a study about cultured T cells differentiation status (Marton et al*.* unpublished data). Thus, authors demonstrated that metabolic potential of T cells is correlated with their differentiation profile evaluated by flow cytometry. Taken together, these data confirmed the appropriate capacity of the evaluated method to measure relevant analytes. Moreover, our experimental observations enabled us to invalidate the hypothesis according to which presence of impurities in assay medium was likely to interfere with analytes measurement. Furthermore, T cell culture conditions with a confluence equal to about 20% resulted in an absence of detected signal in term of OCR or ECAR (data not shown). Altogether, capacity to identify analytes in our experimental model and adequate impurities management permitted to conclude that specificity of the method is conform to expectations.

Excellent accuracy of the Agilent’s provided method was confirmed by calculating the systematic bias of the method associated to Seahorse calibration data. Thus, the bias was largely less than 5% for both O_2_ and pH emission.

Concerning method precision assessment, we first showed that CV are inferior to 15%, the cut-off value of CV associated with a good repeatability. Then, CV values calculated for intermediate precision are all inferior to 30% and are considered as acceptable. In this last setting, despite a thick 95% confidence interval, we can conclude that precision of the method is consistent with expected performance. Yépez et al*.* [21] studied the OCR of adherent primary fibroblasts DHNF using Seahorse technology and demonstrated the precision of the method. The CV for repeatability and intermediate precision evaluation are respectively similar and higher than those we obtained in this study. It indicates that the precision of our method is similar to those of this already published method, although we seeded suspension cells instead of adherent cells. It particularly highlights the fact that the step of cells attachment, in our study, is appropriate and well controlled.

According to the acceptance criteria chosen for the determination coefficient r^2^, i.e. 0.92, we demonstrated that the method is linear for a JURKAT concentration comprised between 25,000–150,000 cells/well. This acceptance criteria is particularly stringent, since, as a rule of thumb, a strong positive correlation has an r-value more than 0.7 [31], demonstrating the analytic relevance of our method. Moreover, the range within which precision is acceptable is comprised between 50,000–150,000 JURKAT/well. Plitzko et al. [28] studied metabolic potential of different cell lines using a Seahorse XF24 analyzer. Authors determined a seeding density likely to induce OCR and ECAR values being within the linear response. They described, at basal state, acceptable linearity of their method and an optimal density seeding of 20,000 and 35,000 cells/well respectively for melanoma cell lines and colon-derived cell line. These values are largely inferior to those we determined for JURKAT cell line for an about 90% confluence. It could be explained by the cell size and the larger place held by adherent cells on the plastic support. It is notable that JURKAT cell line occupy the plastic area with a confluence of about 90% at a concentration of 100,000 cells/well. We could expect that a cell concentration of 150,000 cells/well results in an over-confluence of cells. It is finally not the case, the achieved confluence is quite similar to those previously obtained, because of an obvious cytosolic retraction of the attached JURKAT cells. Similar observations have already been reported by Luciani et al*.* concerning HeLa cell line [32]. Nevertheless, it seems that, at a dose of 200,000 cells/well, JURKAT are not able to contract their cytosol enough to maintain their plastic attachment, inducing a turbidity in wells during the transient micro-chamber formation, likely to interfere with analytes detection. We could assume that the determined range of the method is closely related to confluence: this one could be insufficient under 50,000 JURKAT/well and too important over 150,000 JURKAT/well.

Although the study was not design to go that far, it is of note that a deeper control of JURKAT cell line is required to be used as a reference material in inter-laboratory assays. The reference material should be homogeneous and stable, needing a culture phase after thawing to reach the log phase of cell proliferation; these characteristics are a priori at odds with the prevailing ready-to-use prerequisite for a reference material. Thereby, some steps of validation could remain to be performed in order to provide viable and in log phase-propagating JURKAT cells just after thawing and to validate the appropriate stability of the cell line over cell passages.

Concerning statistical analysis, ICH (Q2) R1 [25] does not establish any criteria for acceptance of the method. In the setting of well-characterized methods, such as chromatography-based assays, the US Food and Drug Administration defines acceptance criteria for pharmaceutical analysis. However, no consensus on criteria for acceptance of exploratory methods exists in the literature. Thus, it could remain difficult to choose relevant acceptance criteria. Although it is relatively easy to meet a consensus for r^2^ minimal cut-off value definition [31], discrepancy in the setting of appropriate CV cut-off definition makes it difficult to address. Methods based on biological analysis are prone to demonstrate a result dispersion higher than those based on chemical dosage. Thereby, CV acceptable threshold should be addressed accordingly. Little [33] even concludes that CV values should always be included in method validation documents as report only and should not form the basis of acceptance criteria. Nonetheless, acceptance criteria for CV were chosen in our work. The cut-off for good precision of 15% was defined according to Cilluffo et al. [34]. It seemed relevant to modulate precision interpretation by adding a cut-off of 30% for acceptable precision, in order to take into account the complexity of the biological-based evaluated method.

Indeed, we demonstrated here the specificity, the precision, and the linearity of the method involving JURKAT cell line as an control material in the setting of T cell metabolic potential assessment and according to ICHQ2 (R1) [25]. Furthermore, we determined the range with acceptable precision and linearity and confirmed the accuracy of the standard provided method.

The use of JURKAT cell line as an experimental control material and the validated method proposed here could be further extended to even better match with potential user scientific inquiries.

Analysis of inter-plate variability could be further controlled, as described by Yépez et al*.* [21], by using a mathematical modeling of the inter-assay variability of the internal control, then integrated in experimentally determined data values. In our assay, we took into account for analysis all three sequential measured values obtained for each basal and post compounds injections. According to Divakaruni et al*.* [26], it could be appropriate to analyze only the minimum or last measurement in the presence of oligomycin because its bioavailability can often result in a time-dependent effect. Similarly, only the maximum measurement after FCCP exposure, due to potentially occurring cell consumption of the molecule, should be considered. This kind of analysis could improve the method precision even more. In our study, we performed all assays using a standard concentration of FCCP, i.e. 1.5 µM. A further characterization of respiratory reserve and capacity could be realized by assaying a dose escalation of FCCP, as advised by Van der Windt et al*.* [35].

Cell resort to OXPHOS and glycolysis could be further investigated using the Seahorse technology by studying the metabolic impact of other compounds. Indeed, the injection of rotenone plus antimycin A, targeting the electron transport chain and by this way reducing OCR to a minimal value, would allow the evaluation of proton leak-linked respiration and a more refined analysis of basal mitochondrial respiration. Rotenone and antimycin A addition would likewise enable to assess qualitative contribution of OXPHOS-related CO_2_ production to ECAR, as described by Divakaruni et al*.* [26]. More specific glycolysis assessment could be implemented by basal ECAR evaluation performed using a glucose non-containing assay medium, then by sequential adding of glucose, oligomycin and 2-deoxy-glucose (2DG) in ports A, B and C of the Seahorse cartridge. Thereby, measuring ECAR just after glucose exposure is likely to refine basal glycolysis analysis and 2DG exposure, by blocking glucose cell uptake and utilization, would contribute to evaluate more slightly non-glycolysis related medium acidification [27]. Taking these considerations into account, further investigations could be performed to extend the validation of the use of JURKAT cell line as a control material for T cell metabolic potential determination.

It is important to note that a relevant control of fitness and proliferative potential of JURKAT cells should be applied. Here, we demonstrated that an appropriate control of passage number and mycoplasma contamination [36], as well as log phase-associated viability [24], seeding and attachment of JURKAT cells are likely to provide acceptable method precision and linearity. Methods and strategies to further normalize XF metabolic data to cellular parameters are available and could be used to improve these key validation parameters and ensure even more accurate results [37]. Total cellular protein assay is a quick and inexpensive method to normalize data but it is also not applicable if there are significant variations in the amount of extracellular matrix protein present among different experimental groups or if plates are coated with protein derived compounds, as poly-D-lysine. Nuclear DNA quantification represents an alternative to protein assessment and is commonly admitted to be correlated linearly with cell number. Cells counting remains the most robust normalization method. It involves cells counting in each well of the microplate via direct imaging or staining cell nuclei.

To conclude, we validated a method using JURKAT cell line as control material to manage the inter-assay variability of a Seahorse technology based-method, in the setting of T cell metabolic potential evaluation. This would allow researchers to compare independent experiments and to improve the robustness of the method. The metabolism analysis methodology developed here presents an adaptation potential to a myriad of scientific inquiries in the setting of T cell metabolism studies, either in term of pathways evaluation or in term of analysis refinement. Our study could represent the first founding element to the use of Seahorse technology to evaluate metabolic potential as a monitoring parameter of ATMP such CAR T cells, in a GMP-compliant environment. Well characterized samples of JURKAT cell line could constitute a reference material to standardize metabolic potential analysis, confirm the accuracy of the method in routine analysis, validate the acceptable level of accuracy across the range and evaluate the reproducibility of the method between different ATMP quality control laboratories.

## Methods

Summarized assay setup is available in Additional file 1: (Fig. S1).

### JURKAT cell line culture and preparation

JURKAT cell line was purchased by the depositary institution DSMZ (ACC 282, DSMZ). It was cultured in RPMI 1640 Glutamax™ medium (72400021, Fisher Scientific) supplemented with 10% Fetal Calf Serum (FCS) (10437036, Fisher Scientific). A master cell bank was cryopreserved 7 passages later. A vial of this bank was used to perform a working cell bank, cryopreserved after 5 supplementary passages. A vial of the JURKAT working cell bank was thawed 3 passages before metabolic evaluation. Master and working cell banks were checked for mycoplasma contamination and master cell bank was authenticated by Short-Term-Repeat (STR) profiling. The day before seeding in the Seahorse culture plate (D_−1_), JURKAT cells are enumerated by Trypan Blue exclusion method and resuspended at a concentration of 0.5.10^6^ cells/mL. At D_0_, JURKAT are enumerated and resuspended in unbuffered prewarmed XF base medium supplemented with glutamine 2 mM, glucose 10 mM and pyruvate 1 mM (103680–100, Agilent Technologies). A minimal acceptable viability of cells of 90% is defined. XF base medium pH is monitored (target value of 7.4). JURKAT cells are seeded in 180 µL/well, according to manufacturer’s instructions and incubated for 1 h in a 37 °C non-CO2 incubator, in a poly-D-lysine (100 µg/mL, 30 µL/well, overnight, 4 °C) (A300-E, Sigma-Aldrich) pre-coated culture plate (Seahorse mini Fluxpak XFe96, 102,601–100, Agilent Technologies). Cell density varied from 200,000 to 25,000 cells/well in a half dilution manner. The cell monolayer is monitored by optic microscopy (Olympus, DP71). The cell area measurement is performed through ImageJ tool, 10 measurement/culture condition are realized; data are presented as the ratio of the mean cell area measured in every seeding conditions on the mean cell area measured in the subconfluent condition (50,000 cells/well).

### XFe96 seahorse assay

At D_−1_, 200 µL/well of distilled water are displayed in the utility plate. The cartridge sensors (Seahorse mini Fluxpak XFe96, 102601–100, Agilent Technologies) are hydrated inside, overnight, in a 37 °C non-CO2 incubator. At D_0_, distilled water is replaced by XF Calibrant (100840–100, Agilent Technologies), cartridge sensors are immersed into the XF Calibrant and incubated for 1 h in a 37 °C non-CO2 incubator. One-hundred and eighty µl of XF Calibrant are added in the four edge-wells in the culture plate. The cover guide is then loaded on the JURKAT-seeded culture plate. Twenty µL of oligomycin (port A) and 22 µL of FCCP (port B) (from Agilent Seahorse XF Cell Energy Phenotype Test Kit, 103325–100, Agilent Technologies) are then displayed, at a final concentration of 1 µM and 1.5 µM, respectively. The utility plate filled with XF Calibrant and capped with the cartridge is positioned in the Seahorse XFe96 analyzer’s tray (Agilent Technologies), and the calibration of the signals generated by all 96 wells is performed. The culture plate is then introduced in the tray and the acquisition program is runned. This program includes a step of equilibration followed by basal, post-oligomycin injection and post-FCCP injection measurements. Each of these three steps comprises three loops of three minutes mixing, two minutes waiting and three minutes measuring. Data are analysed through the Wave 2.6.1 Software (Agilent Technologies).

### Validation of the analytical method: analytical parameters investigated and statistical analysis where applicable

The ICH Q2 (R1) repository is used to guide method validation conception and realization. The method we aimed to validate is assimilated to a content/potency assay. According to ICH Q2 (R1), for this kind of assays, validation criteria are specificity, accuracy, precision, linearity and range. Tests demonstrating an absence of OCR decrease after oligomycin exposure are considered as outliers and are excluded from the analysis.

- Specificity: specificity represents the ability of the method to allow for unambiguous assessment of the analyte in presence of components likely to be present. Specificity is confirmed by comparing OCR and ECAR JURKAT profile obtained at steady state and after oligomycin and FCCP exposure with described profile in the same conditions.
- Accuracy: accuracy expresses the closeness of agreement between the value which is accepted either as a conventional true value or an accepted reference value and the experimental value. Accuracy is confirmed via the calibration step of the sensors, including 96 replicates, corresponding to the 96 wells of a plate. Bias values of the general method are calculated as follows and should be comprised between ± 5% to ascertain an acceptable accuracy:
  $$ Bias = \frac{{mean\;of\;obtained\;values - target\;value}}{{target\;value}} \times 100 $$
- Precision: precision assessment represents the dispersion degree of experimental results and includes repeatability assessment and intermediate fidelity. Repeatability and intermediate fidelity reflect intra-assay and intra-laboratory precision respectively. Fidelity requirements for repeatability evaluation are 6 replicates minimum at the 100% of the test concentration. Repeatability and intermediate fidelity are assessed respectively via 10 replicates and 4 time-delayed experiments of the optimal JURKAT concentration. For each type of precision investigated, standard deviation (SD) is graphically represented, CV and 95% confidence interval (CI 95%) are calculated. CV value is considered as good if inferior to 15%, acceptable if inferior to 30% in the setting of repeatability and intermediate precision, and similarly inacceptable if superior or equal to 30%:
- SD: SD was calculated with the Microsoft Excel 2013 software and corresponds to the formula:
  *SD* = $$ \sqrt {\frac{{\sum {\left( {mean\;of\;obtained\;values\; - \;obtained\;values} \right)^{2} } }}{{sample\;size\; - \;1}}}   $$
- CV:
  $$  CV = \frac{{SD}}{{mean\;of\;obtained\;values}} \times 100  $$
- CI 95%: it is based on Student t distribution, the formula CONFIDENT.T provided by Microsoft Excel 2013 is used, with α = 0.05.
- Linearity and range: a linear relationship should exists across the range of the method. Linearity study requires a minimum of 5 concentrations to be established. The determination coefficient r^2^, γ-intercept, slope of the regression line are submitted. R^2^ acceptance criteria defined is 0.92. Specified range to be considered is from 80 to 120% of the previously determined test concentration. Range is determined as the interval in which appropriate precision is demonstrated for each evaluated concentration. Microsoft Excel 2013 function is used. This one supplies the regression line equation y = ax + b, with a and b corresponding to slope and γ-intercept, respectively, together with the determination coefficient r^2^.

## Supplementary Information

**Additional file 1: Fig S1.** Assay implementation. Summarizing flow-chart of assay setup.

## Data Availability

The datasets used and/or analyzed during the current study are available from the corresponding author on reasonable request.

## Abbreviations

OXPHOS: OXidative PHOSphorylation
m-TOR: Mammalian-Target Of Rapamycin
CAR: Chimeric antibody receptor
ATP: Adenosine TriPhosphate
OCR: Oxygen consumption rate
ECAR: Extracellular acidification rate
GMP: Good manufacturing practices
ICH: International harmonization council
IQC: Internal quality control
ATMP: Advanced therapeutic medicinal product
STR: Short terminal repeats
FCCP: Carbonyl cyanide 4-(trifluoromethoxy)phenylhydrazone
CV: Coefficient of variation
SD: Standard deviation
CI: Confidence interval
2DG: 2-Deoxy glucose
