# Supplementary material for: Validation of a method evaluating T cell metabolic potential in compliance with ICH Q2 (R1)
Source: J Transl Med. 2021 Jan 6;19:21. doi: 10.1186/s12967-020-02672-7 (PMC7789274; doi:10.1186/s12967-020-02672-7)
Supplement: Supplementary file 1 — Additional file 1: Fig S1. Assay implementation. Summarizing flow-chart of assay setup. [file 12967_2020_2672_MOESM1_ESM.pptx]

## Slide 1
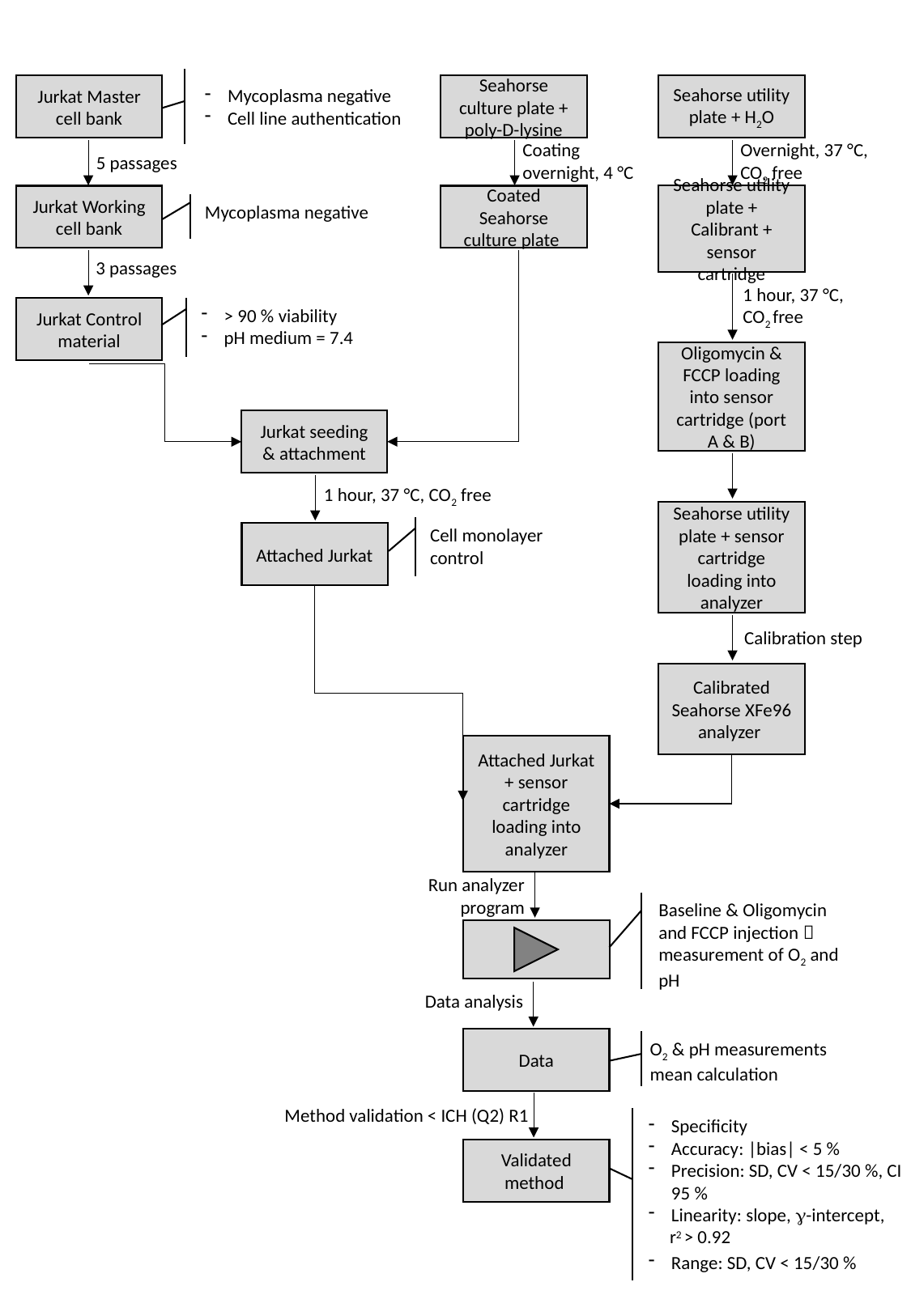

Mycoplasma negative
Cell line authentication
Jurkat Master cell bank
Seahorse culture plate + poly-D-lysine
Seahorse utility plate + H2O
Coating overnight, 4 °C
Overnight, 37 °C,
CO2 free
5 passages
Jurkat Working cell bank
Coated Seahorse culture plate
Seahorse utility plate + Calibrant + sensor cartridge
Mycoplasma negative
3 passages
1 hour, 37 °C,
CO2 free
> 90 % viability
pH medium = 7.4
Jurkat Control material
Oligomycin & FCCP loading into sensor cartridge (port A & B)
Jurkat seeding & attachment
1 hour, 37 °C, CO2 free
Seahorse utility plate + sensor cartridge loading into analyzer
Cell monolayer control
Attached Jurkat
Calibration step
Calibrated Seahorse XFe96 analyzer
Attached Jurkat + sensor cartridge loading into analyzer
Run analyzer program
Baseline & Oligomycin and FCCP injection  measurement of O2 and pH
Data analysis
Data
O2 & pH measurements mean calculation
Method validation < ICH (Q2) R1
Specificity
Accuracy: |bias| < 5 %
Precision: SD, CV < 15/30 %, CI 95 %
Linearity: slope, g-intercept,
 r2 > 0.92
Range: SD, CV < 15/30 %
Validated method
